# Ulipristal acetate before in vitro fertilization: efficacy in infertile women with submucous fibroids

**DOI:** 10.1186/s12958-020-00611-1

**Published:** 2020-05-19

**Authors:** Giuseppe Morgante, Gabriele Centini, Libera Troìa, Raoul Orvieto, Vincenzo De Leo

**Affiliations:** 1 Obstetrics and Gynecology Unit, Department of Molecular and Developmental Medicine, University of Siena, Policlinico “Le Scotte” Viale Bracci, 53100 Siena, Italy; 2 Department of Obstetrics and Gynecology, Chaim Sheba Medical Center, Tel-Hashomer, 52621 Ramat Gan, Israel; 3 Sackler Faculty of Medicine, Tel Aviv University, 39040 Tel Aviv, Israel; 4 The Tarnesby-Tarnowski Chair for Family Planning and Fertility Regulation, Sackler Faculty of Medicine, Tel-Aviv University, 39040 Tel Aviv, Israel

**Keywords:** Submucous fibroids, Uterine leiomyomas, Ulipristal acetate, In vitro fertilization, Assisted reproductive technology

## Abstract

**Background:**

The presence of submucous fibroids strongly impacts on IVF results, therefore, these patients should be considered for surgical or medical treatment. The aim of this study was to assess the role of Ulipristal acetate (UPA), a selective progesterone receptor modulator, in restoring uterine cavity deformation due to submucous fibroids, in infertile patients attempting an IVF treatment. The secondary study outcome was to evaluate the impact of preconception UPA treatment on rate of biochemical pregnancy, ongoing pregnancy, and live birth compared to a control group without fibroids.

**Methods:**

Infertile patients with submucosal fibroid (Type 1 and Type 2 according to FIGO classification) were enrolled in the study as fibroids group and received 1 to 3 treatment cycles of UPA, according to their response, as reflected by fibroid volume reduction and restoration of normal uterine cavity. Patients in control group were randomly selected from a general IVF cohort by a ratio of 2:1 with fibroids group, matched by age, BMI, type and cause of infertility and antral follicle count. The impact of UPA on fibroids volume reduction was evaluated. IVF outcome was compared between groups.

**Results:**

Twenty-six patients underwent UPA treatment revealed a mean volume reduction of their fibroids of 41%. A total of 15 (57.6%) biochemical pregnancy were obtained, resulting in 13 (50%) ongoing pregnancy and 9 (34.6%) healthy babies were already delivered. Similar results were obtained in control group.

**Conclusion:**

Restoration of normal uterine cavity by UPA treatment prior to IVF treatment avoids surgery and establishes a pregnancy rate comparable to a control group without fibroids.

## Background

Uterine leiomyomas, or fibroids, are the most common form of benign uterine tumors occurring in 20–40% of women of reproductive age [1]. They are hormone-sensitive, smooth-muscle tumors with a wide heterogeneity in composition, size and number [2].

In the majority of cases, fibroids are asymptomatic, the diagnosis is incidental and need no intervention. However, third of cases represent with a variety of symptoms, depending on their location and size, and require treatment. The most common symptom is abnormal uterine bleeding, usually excessive menstrual bleeding with subsequent anemia, which could be life-threatening. Other symptoms include pelvic pressure, bowel dysfunction, urinary frequency and urgency, urinary retention, low back pain, constipation, dyspareunia and obstetrics complications [2].

Infertility and recurrent miscarriages may also be symptoms of fibroids. Their anatomical location, specifically submucous and intramural fibroids, seems to be the most relevant factor affecting fertility and fertility treatments [1, 3, 4].

Fibroids can impair fertility through several possible mechanisms including: alteration of the local anatomy (anatomic distortion of the uterine cavity) with subsequent endometrial function modification [3]; functional changes, such as increased uterine contractility and impairment of the endometrial and myometrial blood supply [1]; and changes to the local hormone milieu which could impair gamete transport and/or reduce blastocyst implantation [5]. The type of treatment is guided by fibroid’s characteristics (size, number and location), patient’s age and whether the women desires to preserve fertility or not.

Submucous fibroids (Type 1 and 2 according to FIGO leiomyoma sub-classification system) [6] distorting the uterine cavity, negatively impact implantation rates, pregnancy outcomes and IVF treatments success [1, 3, 4, 7, 8]. Therefore, before starting IVF procedure, the resection of submucosus fibroids is strongly recommended [9]. Treatment of fibroids is mainly surgical and the gold standard for submucosal fibroids is hysteroscopic myomectomy, enhancing conception and live births with a pregnancy rate in infertile women ranging from 16 to 76.9% [1, 7, 9, 10].

Beside the surgical eradication, several medical therapies are now available. One of which is Ulipristal acetate (UPA), a selective progesterone receptor modulator (SPRM). UPA is effective in controlling excessive bleeding, reducing fibroids volume and has been proposed as a pre-operative treatment in those women with symptomatic myomas undergoing surgical therapy [11, 12]. Data on pregnancy achievement after UPA treatment are inconsistent and mainly based on case reports and small series, but it seems to enhance the chance of conception [13, 14].

Luyckx et al. report the first series of pregnancies achieved after UPA treatment, with a pregnancy rate of 71% in the group of patients wishing to conceive. They also describe pregnancies obtained after UPA treatment for fibroids in women who did not undergo surgery [14]; however only two patients conceived without surgery and this contributes to the weakness of the published results. Recently, the first case of infertile advance-age patient with large intramural fibroid, who conceived by IVF treatment following a course of Ulipristal was reported [15]. The patient underwent two fresh fertility preserving IVF cycles, with cryopreservation of 9 day-3 embryos, followed by a 12 weeks course of UPA (5 mg per day) and a subsequent frozen-thawed embryo transfer with her own previously cryopreserved embryos.

Prompted by the aforementioned information we aimed to evaluate the efficacy of UPA in avoiding surgery and restoring uterine cavity deformation due to submucous fibroids, in infertile patients attempting an IVF treatment. The secondary outcome was to evaluate the impact of pre-IVF UPA treatment on number of oocyte retrieval and embryos obtained, biochemical pregnancy rate, ongoing pregnancy rate, live birth rates, compared to a control group without fibroids.

## Materials and methods

A prospective study was conducted at “Santa Maria alle Scotte” University Hospital, Department of Molecular and Developmental Medicine and it was approved by the ethical committee of the Siena University under the ID 10818_2017 clinical protocol.

Consecutive patients with uterine fibromatosis who underwent their first IVF cycle between March 2017 and March 2018 were included in the study. The inclusion criteria in the fibroids group were: age between 20 and 38 years; regular menstrual cycles of 25–35 days; basal FSH less than 12 IU/L (cycle day 2–5); total antral follicle count of 10–25 follicles; infertility resulting from tubal factors; unexplained infertility; presence of both ovaries; IVF cycle followed by fresh embryo transfer.

The first selection criterion in the fibroids group was the presence of a submucosal fibroid with more than 3 cm diameter, which cannot be treated with one step hysteroscopic approach [9]. Only those classified as Type 1 to Type 2, according to the FIGO classification [6] and distorting the uterine cavity were included.

The exclusion criteria were: obesity (patient BMI > 30 Kg/m^2^); more than 2 fibroids; history of myomectomy; endometrial lesions (polyps, endometrial hyperplasia, and intrauterine adhesions); uterine malformations (septum, unicornuate or bicornuate uterus); sonographic features of endometriosis and/or adenomyosis; previous surgery for endometriosis; history of pelvic inflammatory disease; polycystic ovarian syndrome; previous surgery for infertility; chromosomal abnormality of male or female partner.

Other major comorbidities such as diabetes, hypertension, bowel chronic diseases, rheumatologic diseases or male infertility were also considered as exclusion criteria. All medical conditions (including obesity) that can interfere with the female fertility rate have been excluded, in order to eliminate these possible confusing factors. Only female fertility was considered.

In an attempt to demonstrate that women with submucosal myomas, following treatment with Esmya before IVF, achieved fibroid regression and restoration of the uterine cavity, as reflected by their success rate during IVF, we chose to compared them to healthy women without fibroids. This control group included women with normal uterus that underwent IVF treatment during the same period and were randomly selected from the same database, matched by age, type of infertility (primary and secondary), cause of infertility (Tubal, Ovarian or Unexplained factors), and antral follicle count, in a ratio of 2:1 with fibroids group. The same selection criteria used for UPA treatment selection were applied to the control group.

All the women of the fibroid group underwent pre-treatment transvaginal ultrasound in which the size of fibroids (the three major diameters) were recorded and a sonohysterography to assess the cavity distortion was performed. After confirming the diagnosis, UPA tablets 5 mg (Esmya, Gedeon Richter, Italy) was prescribed and the patients started therapy at the beginning of the next menstrual cycle. Each patient was asked to take UPA at the dose of one tablet every day for 84 days (first cycle of treatment) up to 3 cycle of treatment, with a full menstrual cycle wash-out between two consecutive cycles; a blood sample was collected monthly to evaluate the liver enzymes profile and test UPA toxicity. This treatment and management are part of our standard clinical practice.

Follow-up visits were carried out at the end of every cycle of UPA therapy: fibroids size was measured by transvaginal ultrasound and a sonohysterography was repeated. A normal uterine cavity with no remaining myomas submucosal portion at sonohysterography was considered the condition allowing to proceed to ART; otherwise, only if a reduction of the volume was detected, an additional UPA cycle was proposed to the patient. In case of no change in fibroid volume the patient was withdrawn from the study and referred to surgery.

The time lapse between Ulipristal therapy and IVF treatment was a menstrual cycle. Menses were synchronized with combined oral contraception pills and ovarian hyperstimulation was carried out from the second day of the menstruation with a standard start dose of 225 IU of urofollitrophin hormone (uFSH).

The dose was adjusted based on follicle measurements and hormonal evaluation of estradiol (E2) and progesterone (P) at the first ultrasound examination on day 6 of the cycle and subsequently every 2–3 days. When follicles reached a mean diameter of 14 mm, GnRH antagonist was started and continued throughout the stimulation period.

Once at least one follicle reached a diameter of > 18 mm and two additional follicles reached a diameter of > 16 mm, 250 mcg of r-hCG (Ovitrelle; Merck Serono, Germany) was administered to trigger ovulation, and 34–36 h later oocytes were retrieved.

A maximum of two cleaved-embryos or blastocysts were transferred 2–5 days after oocyte retrieval.

Vaginal capsules of micronized 200 mg progesterone (three times/day) were administered from the day of oocyte retrieval and continued for at least 14 days after embryo transfer. This ART procedure refer to both control and fibroid group.

Biochemical pregnancy was defined as transiently positive β-hCG level not associated with the development of an embryo, while ongoing pregnancy was referred as a viable intrauterine pregnancy of at least 12 weeks duration confirmed on ultrasound scan.

Medical history of all women was collected from our electronic database and data about previous pregnancy or surgery, ovarian stimulation, oocyte retrieval and ART details were recorded.

Plasma concentration of FSH and AMH were measured using Access Immunoassay System (Beckman Coulter, Milan, Italy); for progesterone and estradiol were used Immunolite 2000 system Kits (Siemens, Los Angeles, CA, USA). The samples were analysed twice with two dilutions. For each test, controls at low, medium and high concentration were included. The dosing limits were 0,1 ng/ml for progesterone, 15 pg/ml for estradiol, 0,2 mUI/ml for FSH and AMH. Plasma liver enzymes was determined by an enzymatic assay (Bristol, Paris, France). The methods used are highly specific for each hormone and have low cross-reactivity (b.0.5%) with other hormones or drugs present in the samples.

### Statistical analysis

The Student’s t-test was used to compare means of the two groups for normally distributed continuous variables, and paired sample t-test was performed. The Mann-Whitney U test was used when continuous variables did not follow a normal distribution. The chi-squared test or Fisher’s exact test, where appropriate, were used for comparisons of categorical variables. The data are presented as mean, standard deviation (SD) or as percentages. Statistical significance was set at a *p* value < 0.05. All statistical analyses were performed using the Graph Pad Prism 6 software.

## Results

During the study period a total of 40 patients with infertility and a diagnosis of submucosal fibroid (Type 1 and Type 2) were referred to our fertility clinic, of whom 27 were eligible and therefore enrolled in the study as fibroids group and received 1 to 3 cycle of UPA treatment, accordingly to the volume reduction and the effect of the fibroid on the uterine cavity. Patients’ characteristics are showed in Table 1.

**Table 1:** Patients’ characteristics: anthropometric data, parity and number of UPA cycles have been reported

Patients’ characteristics	Fibroids group (N 27)	Control group (N 54)	*P* value
**Age (years)**	33.72 ± 1.77	33.83 ± 0.39	ns
**Body mass index (Kg/m**^**2**^**)**	23.44 ± 1.86	23.67 ± 0.25	ns
**Nulliparous**	20 (74)^a^	38 (70)^a^	ns
**Primiparous**	7 (26)^a^	16 (30)^a^	ns
**Previous miscarriage**	14 (52)^a^	25 (46.3)^a^	ns
**Number UPA cycle**	1.8 ± 0.58	Na	
**Patients with one fibroid**	15 (55)^a^	Na	
**Patients with two fibroids**	12 (45)^a^	Na	

Data are expressed as Mean ± Standard Deviation

*Na* not applicable, *Ns* not significant

^a^Data are expressed as absolute number (percentage)

Mean patients’ age was 33,7 years (range 30–36). The mean number of fibroids per patient was 1.4; 15 patients had one fibroid while 12 had 2 fibroids (Table 1). The mean diameter of the fibroids distorting the cavity was 5.5 cm (ranging from 3.7 to 6.3 cm).

**Fig. 1:**
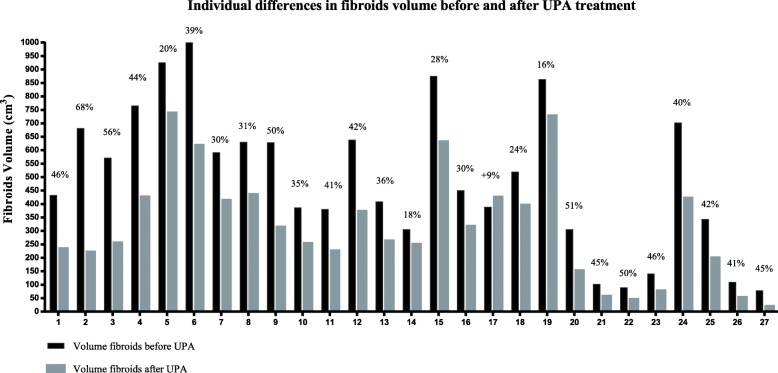
Individual differences in fibroids volume before and after UPA treatment. UPA treatment led to a mean reduction in volume of the myoma impacting the cavity of 41% ± 13.37. The volume before treatment was 399.17 ± 285.20 ml and become 257.02 ± 203.83 after UPA cycles. The volume of myoma nodule was calculated as 1/6 π × L1 × L2 × L3 where L1, L2, and L3 are the three diameters of the nodule that are at right angles to each other. Individual differences in fibroids volume after treatment and the percentage of volume reduction for each patients were reported. The percentage of reduction was always significant. Only one patient did not respond to UPA treatment and showed a volume augmentation of 9.2%

The patients received a mean of 1.8 UPA treatment cycles leading to a mean 41% ± 13.37 reduction in the fibroid volume (range 16 to 68%) (Fig. 1). The volume before treatment was 399.17 ± 285.20 cm3 and become 257.02 ± 203.83 cm3 after UPA cycles. The volume of myoma nodule was calculated as 1/6 π × L1 × L2 × L3 where L1, L2, and L3 are the three diameters of the nodule that are at right angles to each other. Only one patient did not respond to UPA treatment and showed a volume augmentation of 9.2% and was therefore excluded from the study and referred to surgery after the first 3-month UPA treatment cycle.

Biochemical parameters are reported in Table 2: after UPA treatment patients showed a significant improvement in hemoglobin and hematocrit Follicle-stimulating hormone (FSH) values significantly increased, with no changes in anti-mullerian hormone (AMH) values. No differences are recorded between fibroids group after UPA treatment and the control group (Table 2). Amongst the 26 patients who underwent ovarian stimulation for IVF, a mean of 4.4 (2–7) oocyte per patient were collected; 3.4 (1–5) embryos were obtained and a maximum of 2 embryos were transferred. A total of 15 (57.6%) biochemical pregnancy were obtained resulting in 13 (50%) ongoing pregnancy and 9 (34.6%) healthy babies were already delivered. No cases of fetal malformation, stillbirth or neonatal death were recorded. When compared with the control group the same results were obtained (Table 3).

**Table 2:** Patients’ biochemical parameters: hormonal and hemoglobin values have been reported. The liver enzyme profile was also analyzed to assess UPA toxicity

Patients’ biochemical parameters	Fibroids group “before UPA”	Fibroids group “after UPA”	Control group	*P* value
**FSH (mUI/ml)**	9.83 ± 1.14	10.04 ± 1.26	10.24 ± 0.23	0.0034^a^ ns^b^
**AMH (mUI/ml)**	1.15 ± 0.26	1.11 ± 0.23	1.16 ± 0.05	ns^a^ ns^b^
**Hb (g/dl)**	11.06 ± 0.66	11.51 ± 0.50	11.76 ± 0.12	0.0001^a^ ns^b^
**Hct (%)**	36.58 ± 1.47	37.11 ± 1.19	37.27 ± 0.20	0.0027^a^ ns^b^
**GOT (UI/l)**	16.45 ± 0.76	17.01 ± 0.56	17.59 ± 0.66	ns^a^ ns^b^
**GPT (UI/l)**	12.16 ± 0.45	13.58 ± 0.55	13.34 ± 0.75	ns^a^ ns^b^
**Gamma glutamyl transferase (UI/l)**	5.21 ± 0.15	6.01 ± 0.19	5.65 ± 0.23	ns^a^ ns^b^
**Alkaline Phosphatase (UI/l)**	43.41 ± 1.25	44.13 ± 1.05	44.21 ± 0.78	ns^a^ ns^b^

Data are expressed as Mean ± Standard Deviation

*FSH* follicle-stimulating hormone, *AMH* anti-mullerian hormone, *Hb* hemoglobin, *Hct* hematocrit, *Ns* not significant

^a^between fibroids group before and after UPA treatment

^b^between fibroids group after UPA and control group

**Table 3:** IVF and neonatal outcomes

IVF outcomes	Fibroids group (N 26)	Control group (N 54)	*P* value
**Oocyte retrieval**	4.44 ± 1.29	4.27 ± 0.27	ns
**Number Embryos obtained**	3.44 ± 1.20	3.27 ± 0.23	ns
**Biochemical pregnancy**	15 (57.6)^a^	33 (61.1)^a^	ns
**Ongoing pregnancy**	13 (50)^a^	27 (50)^a^	ns
**Healthy babies delivered**	9 (34.6)^a^	20 (37)^a^	ns

Data are expressed as Mean ± Standard Deviation

*Ns* not significant

^a^Data are expressed as absolute number (percentage)

Within the fibroids group, no differences in the amount of volume fibroids reduction were observed between patients who conceived, had biochemical/ultrasound diagnosis of pregnancy or childbirth, and those who did not (Table 4). No adverse effects or liver enzymes alteration were recorded during the study period (Table 2).

**Table 4:** Within the fibroids group, characteristics relating to fibroids are reported. Fibroids volume reduction after UPA treatment was compared between patients who conceived and those who did not

	Patients who conceived	Patients who did not conceived	*P* value
**Number of UPA cycle**	1.9 ± 0.51	1.7 ± 0.61	ns
**Number of fibroids**	1.36 ± 0.50	1.54 ± 0.52	ns
**Fibroids Localization**			
**-anterior**	42%	36%	ns
**-posterior**	38%	41%	ns
**-lateral**	20%	23%	ns
**% of fibroids volume reduction**	41.4 ± 12.44	39.51 ± 14.48	ns

Data are expressed as Mean ± Standard Deviation

*Ns* not significant, *UPA* Ulipristal acetate

## Discussion

Our study shows that restoration of normal uterine cavity prior to IVF treatment by UPA therapy may avoid surgery and establishes a pregnancy rate comparable to a control group without fibroids. The role of progesterone and its receptors has been extensively studied during the last years as being decisive in promoting the growth of uterine fibroids and has stimulated interest in modulating the progesterone pathways [16].

Selective progesterone receptor modulators (SPRMs) are drugs that exert agonistic or antagonistic effect on progesterone receptors and can modulate progesterone effect on different tissues [17]. UPA is a SPRM that block progestogen activity and is effective in reducing uterine fibroids volume. This effect lasts over time, without major side effects [17]. In line with previous publications, in our series there was a mean volume reduction of 41% and only one patient was referred to surgery because no fibroid size reduction was detected.

The advantages of UPA are rapid reduction of amount of bleeding in most cases and a significant reduction in fibroid volume [9]. Christopoulos et al. demonstrated that the presence of fibroids not distorting uterine cavity negatively affect clinical pregnancy (odds ratio, OR 0.62; 95% confidence interval, 95% CI 0.41–0.94) and live birth rates (OR 0.58; 95% CI 0.48–0.78) in patients undergoing their first IVF/ICSI cycle [18]. The presence of submucous fibroids strongly impacts on IVF results, therefore, this patient should be considered for surgical or medical treatment [4]. Data on UPA exposure before IVF is limited, except for some case reports focused on intramural fibroids [15, 18, 19]. Wdowiack et al. reported a case of pre-treatment with UPA before an ICSI procedure ending with conception and vaginal delivery of a baby [19]. Lo Monte et al. report a case of multiple uterine fibroids with two fibroids distorting the uterine cavity, who were treated with 3 months UPA prior to hysteroscopic myomectomy and followed for three more months. Nine months after a second cycle of UPA the patient underwent an IVF treatment [20]. Moreover, since the major disadvantage of myomectomy is the need for an optimal waiting period between surgery and subsequent fertility treatment [21].

Orvieto et al. has suggested that while counselling an advance-age patient with prominent intramural fibroid, the treatment of choice should be 1–3 IVF cycles, aiming to cryopreserve 5–10 embryos, followed by a 12 weeks course of Ulipristal and a subsequent FET with her own previously cryopreserved embryos [15].

Our study have some limitations. The high success rate recorded in our sample, concerning the restoration of uterine cavity can be explained by the following factors: low number of patients, young age, and obesity as an exclusion factor. These features could improve response to therapy and may have limited the generalizability of our findings. Moreover, our healthy control group was chosen in an attempt to demonstrate that women with submucosal myomas, when treated with Esmya before IVF, achieved IVF success rate comparable to healthy women without fibroids. An ideal control would be women with previous myomectomy undergoing IVF, which unfortunately we could not achieve.

The present study is the first case-series, where UPA has been used in patient with fibroids distorting the uterine cavity prior to IVF treatment, demonstrating an ongoing pregnancy rate of 50%, comparable to that obtained in patients without fibroids. No differences in the amount of volume fibroids reduction were observed between patients who conceived and those who did not. Getting pregnant is the result of multiple factors, not only mechanical, but also hormonal, endometrial, vascular and inflammatory. UPA treatment is important not only because it reduces the size of the fibroids and their impact on the uterine cavity but allows to reduce the inflammatory state associated with these lesions. On the one hand restoring the correct anatomy of the uterus, and on the other hand reducing the inflammatory state are fundamental goals for achieving pregnancy in these women.

Furthermore, we didn’t observe any complication during pregnancy, related to excessive growth of the fibroid. Apoptosis and the sustained decrease in myoma size seen after UPA treatment could explain the absence of regrowth during pregnancy, despite high levels of circulating progesterone [14].

Indeed, in the case of pregnancy the presence of myomas -in particular of myomas that distort the uterine cavity and larger intramural myomas- has been linked to an increased risk of spontaneous abortion, fetal malpresentation, placenta previa, preterm birth, cesarean section, and peripartum hemorrhage [22]. Clinical experience and observational studies suggest that fibroid treatment may improve the outcome of pregnancy [22, 23].

Pre ART UPA treatment does not impair embryos quality or foetal morphology and the subsequent pregnancy did not affect fibroid size [19].

As described by Donnez in 2016, in case of fibroids greater than 3 cm, a preoperative treatment is advisable [9]. Pre-surgical UPA treatment for large and complex fibroid induces its shrinkage and increases the rate of complete resection with a shorter surgical procedure, allowing the possibility of one-time hysteroscopic resection [23, 24].

Our case-series suggest the pre-ART UPA treatment, as a possible alternative to surgery, with a reasonable pregnancy rate and avoiding possible surgical complications [25].

In conclusion, the presence of submucous fibroids strongly impacts on IVF results, therefore, this patient should be considered for surgical or medical treatment. UPA therapy is efficient in restoring uterine cavity deformation and improving subsequent IVF outcome. Avoiding surgery is also crucial in infertile women. Further large randomized controlled studies are needed to confirm our observation and to further define patient selection criteria. These will aid both fertility specialists’ counselling and their patients in tailoring the correct approach to submucosal fibroid, optimizing the results without losing time.

## Data Availability

The datasets used and/or analysed during the current study is available from the corresponding author on reasonable request.

## Abbreviations

AMH: Anti-mullerian hormone
ART: Assisted reproductive technology
β-hCG: Beta human chorionic gonadotropin
BMI: Body mass index
CI: Confidence interval
E2: Estradiol
FIGO: International Federation of Gynecology and Obstetrics
FSH: Follicle-stimulating hormone
GnRH: Gonadotropin-releasing hormone
Hb: Hemoglobin
Hct: Hematocrit
ICSI: Intracytoplasmic sperm injection
IVF: In vitro fertilization
OR: Odds ratio
P: Progesterone
SD: Standard deviation
SPRM: Selective progesterone receptor modulator
r-hCG: Recombinant human chorionic gonadotropin
uFSH: Urofollitrophin hormone
UI: International unit
UPA: Ulipristal acetate
